# The association of resveratrol and AFPI did not enhance the cryoresistance of ram sperm

**DOI:** 10.1590/1984-3143-AR2023-0159

**Published:** 2024-02-12

**Authors:** Viviane Lopes Brair, Lucas Francisco Leodido Correia, Nathalia Oliveira Barbosa, Rachel Ferreira Braga, Augusto Ryonosuke Taira, Andreza Amaral da Silva, Felipe Zandonadi Brandão, Rodolfo Ungerfeld, Joanna Maria Gonçalves Souza-Fabjan

**Affiliations:** 1 Faculdade de Veterinária, Universidade Federal Fluminense, Niterói, RJ, Brasil; 2 Instituto de Veterinária, Universidade Federal Rural do Rio de Janeiro, Seropédica, RJ, Brasil; 3 Departamento de Biociencias Veterinarias, Facultad de Veterinaria, Universidad de la República, Montevideo, Uruguay

**Keywords:** antioxidant, cryoprotectants, sheep, slow-freezing, spermatozoa

## Abstract

Cryoprotectants are required to reduce damage caused to the cells due to low temperatures during the cryopreservation. Antifreeze proteins (AFP) have a well-known role in cell membrane protection, while resveratrol is a potent antioxidant. This study assessed the effect of the association of resveratrol concentrations and AFP I in a ram semen extender. Pooled semen of four rams was allocated into six treatments in a factorial arrangement: (CONT, only the semen extender); only AFP I (ANT: 0.1 µg/mL of AFP I), only resveratrol, one treatment with two levels (10 µM/mL or 50 µM/mL of resveratrol); and two treatments with the interactions, with one AFP I and one of the two levels of resveratrol (0.1 µg/mL of AFP I with 10 µM/mL resveratrol; 0.1 µg/mL of AFP I with 50 µM/mL resveratrol). No interaction between factors was observed on sperm kinetics, plasma membrane integrity, hypo-osmotic test, and mitochondrial activity parameters. There was a high probability (*P* = 0.06) of reducing sperm cells with functional membrane percentage in the hypo-osmotic test and increasing the percentage of sperm with high mitochondrial activity (*P* = 0.07) was observed in AFP presence. An interaction of AFP and resveratrol was observed in non-capacitated sperm (*P* = 0.009), acrosomal reaction (*P* = 0.034), and sperm binding (*P* = 0.04). In conclusion, the association of resveratrol and AFP did not improve the quality of frozen-thawed semen and even promoted deleterious effects compared to their single addition in the semen extender. The supplementation of 50 µM/mL of resveratrol improved the outcomes of frozen-thawed ram sperm, being a potential cryoprotectant.

## Introduction

Semen cryopreservation is used to manage and preserve male gametes in domestic animals, preserve endangered species, or accelerate the rate of genetic improvement (Arav and Saragusty, 2018). However, cryopreservation processes induce several cryoinjuries mainly provoked by the formation of ice crystals, induction of osmotic stress, and generation of excessive reactive oxygen species (ROS). These alterations often lead to acrosome damage, depolarization of the mitochondrial membrane, cryo-capacitation, reduction of fertilizing capacity, and modifications in plasma membrane permeability and mitochondrial membrane fluidity, which cannot be repaired due to sperm cell structure and physiology (Upadhyay et al., 2021).

In particular, only 40-60% of the ram spermatozoa remain motile after thawing, and only 20-30% maintain their biological function (Lv et al., 2019a). Therefore, several new cryoprotectants have been tested to reduce the injuries caused by freezing-thawing. In this context, some molecules, such as antifreeze proteins (AFPs), protect the membranes and, when added to the extender, have important benefits in the cryopreservation of ram (Payne et al., 1994; Correia et al., 2021a), rabbit (Nishijima et al., 2014), and buffalo (Qadeer et al., 2016) spermatozoa. The AFPs induce thermal hysteresis and reduce ice crystal formation during cryopreservation (Kim et al., 2017). There are four main types of fish-derived AFP, varying according to their affinity in ice crystal binding faces: type I, II, III, and Glycoprotein (AFGP) (see review Correia et al., 2021b). The addition of AFP I, more precisely at 0.1 µg/mL, to ram semen before the freezing-thawing process resulted in a greater percentage of motile sperm (Payne et al., 1994), leading to improvement in sperm kinetics, plasma membrane integrity, and final percentage of normal sperm cells after thawing (Correia et al., 2021a). Moreover, the addition of 0.1 µg/mL of AFP I did not affect mitochondrial activity, capacitation, and lipoperoxidation (Correia et al., 2021a), suggesting its activity could not reduce the negative effects of ROS caused by cryopreservation.

A physiological concentration of ROS molecules is required for sperm capacitation, acrosome reaction, and zona binding events. However, elevated levels of ROS during cryopreservation induce oxidative stress, detrimentally impacting sperm motility, DNA integrity, and overall sperm competence (Gualtieri et al., 2021). ROS are mainly represented by several scavengers, but hydrogen peroxide (H_2_O_2_) is the main responsible for inducing greater DNA damage and genome alteration during cryopreservation. Thus, the increase of H_2_O_2_ leads to an increase in lipoperoxidation that is related to DNA instability, chromatin integrity, and semen quality in ram sperm (Peris et al., 2007). Therefore, several antioxidants have been used, combined or not with other molecules in semen extenders (Gualtieri et al., 2021). In this context, determining if there is an association between the effects of AFP and antioxidants can open interesting possibilities to improve the results of freezing-thawing sheep sperm.

Resveratrol (3,5,4’-trihydroxystilbene) is a polyphenol with an antioxidant function found in numerous plant species and red wine (Harikumar and Aggarwal, 2008). Resveratrol has been applied in several studies in mammalian reproduction (Pasquariello et al., 2020). In ram semen, the use of resveratrol was related to positive effects on sperm motility in semen stored at 5 ºC (Sarlós et al., 2002), and when added to the cryopreservation extender at concentrations of ~20 to 90 µM/mL it enhanced the mitochondrial membrane potential in frozen-thawed sperm (Silva et al., 2012). Moreover, 10 to 100 µM/mL of resveratrol reduced ROS, promoted greater plasma membrane integrity (Lv et al., 2019b), and reduced apoptosis in goat semen (Falchi et al., 2020). The same concentrations induced lower DNA fragmentation, lipid peroxidation, higher fertilization capacity, and antioxidant activity in buffalo semen (Longobardi et al., 2017; Ahmed et al., 2020). However, it is still unclear whether resveratrol can improve ram sperm motility. Therefore, this study hypothesized that the addition of resveratrol associated with AFP I enhances rams’ sperm cryotolerance and kinetics. Thus, this study aimed to determine if the association between different concentrations of resveratrol and a fixed concentration of AFP I enhances the quality of post-thawed ram sperm.

## Methods

### Chemical reagents

All reagents were acquired from Sigma Chemical Co (St. Louis, MO, USA), except the AFP I acquired from A/F Protein Inc (Waltham, MA, USA), and were diluted according to the manufacturer’s instructions.

### Ethics approval, experimental conditions, and animals

The Ethics Committee for Use of Animals of Universidade Federal Fluminense approved this study (#3696250121). Semen collection, pre- and pos-thawing evaluations were conducted at Unidade de Pesquisa em Caprinos e Ovinos (UniPECO), in Cachoeiras de Macacu, Rio de Janeiro, Brazil (22º 27′ S, 42º 39’ W) during the late breeding season (August and September). Samples were collected from four healthy adult Santa Inês breed rams aged 3 to 4 years old, with a body condition score of 3 to 4 (scale 1-5), that previously underwent andrological and clinical assessment (CBRA, 2013). Animals remained in partial confinement and received concentrate and Napier grass (*Pennisetum purpureum*) at night, according to nutritional requirements and had free access to pasture during the day, with free access to water and mineral salt.

### Experimental design

**Figure 1:**
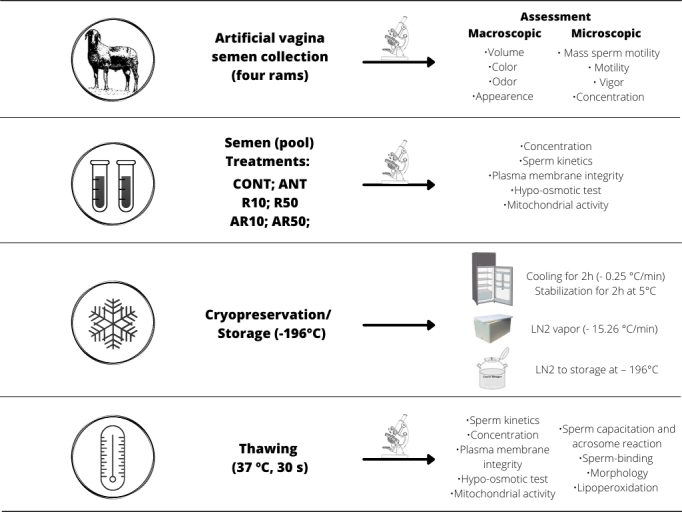
Experimental design scheme: CONT) control containing only extender; ANT) added of AFPI; R10) 10 µM/mL resveratrol; R50) 50 µM/mL resveratrol; AR10) AFP I with 10 µM/mL resveratrol; AR50) AFP I with 50 µM/mL resveratrol. The concentration of AFP I was: 0.1 µg/mL.

Semen was collected in an artificial vagina from each ram four times with intervals of 12 h to homogenize the status of the animals, and then the rams had a sexual rest of one day before beginning the study. For cryopreservation, semen collection was carried out only once a day, in the morning, following the same order of rams for six days. The ejaculates were microscopically evaluated (mass sperm motility, motility, vigor, concentration) according to CBRA (2013). Each ejaculate was assessed and those with ≥ 70% of motile sperm were pooled. The total volume of each ejaculate was used in the preparation of the pool, to minimize individual influences. The sperm pool concentration was assessed by Neubauer chamber, and semen was allocated into six experimental treatments in a factorial arrangement, with a final concentration of 100 × 10^6^ spermatozoa per straw (0.25 mL). The factors were the addition of AFP I (0.0 or 0.1 µg/mL) and/or resveratrol (0, 10, or 50 µM/mL) to the semen extender. Therefore, the treatments were: control (CONT, only the semen extender); only AFP I treatment, with only one level (ANT: 0.1 µg/mL of AFP I); only resveratrol (R10: 10 µM/mL resveratrol or R50: 50 µM/mL resveratrol); and the two interactions treatments, with one level of AFP I and one of the two levels of resveratrol each (AR10: 0.1 µg/mL of AFP I with 10 µM/mL resveratrol or AR50: 0.1 µg/mL of AFP I with 50 µM/mL resveratrol). Immediately after dilution, sperm kinetics, plasma membrane integrity, hypo-osmotic test, and mitochondrial activity analyses were performed in one single sample for each treatment daily. At the same time, semen was cryopreserved by a slow freezing technique. Immediately after thawing, all the previous analyses were performed, and sperm capacitation and acrosome reaction, morphology, sperm binding, and lipoperoxidation were also assessed (Figure 1). All evaluations were performed by the same technician and followed the same criteria.

### Cryopreservation media and thawing process

Semen cryopreservation was performed according to each treatment, using TRIS egg yolk extender [(3.63 g TRIS, 0.50 g fructose, 1.99 g citric acid, 15 mL egg yolk, 100.000 IU penicillin, 100 mg streptomycin, 5% glycerol, glass-distilled water to 100 mL (Correia et al., 2021a)], with pH 7.4 and 1180 mOsm/kg. The extender was divided into aliquots for each treatment and kept frozen until daily use. After semen dilution, the plastic straws of 0.25 mL were filled, identified, and sealed (polyvinyl alcohol). Afterward, straws were placed inside a warm metal basket in a heating plate at 37 °C and were cooled for 2 h in a refrigerator until it reached 5 °C (-0.25 °C/min) and maintained in the refrigerator for two more hours for stabilization. Immediately after the refrigerator step, the metal basket was submerged in liquid nitrogen vapor for 10 min until reaching -140 °C (-15.26 °C/min) and immersed in liquid nitrogen at -196 °C (Jha et al., 2019). Thawing was performed in a water bath at 35 °C for 30 s.

### Sperm kinetics

The sperm kinetics was analyzed by Computer-Assisted Semen Analysis (CASA) using the SCA system (Sperm Class Analyzer Microptic, Nikon Eclipse Ci – Tokyo, Japan), after software configuration for ram sperm (Olivares et al., 2017). The parameters were set as the head dimension between 18 and 60 μm^2^; static - curvilinear velocity (VCL) lower than 10 μm/s; slow - between 10 and 45 μm/s; medium - between 45 and 75 μm/s; fast - more than 75 μm/s; progressive motile sperm - straightness (STR) of sperm cells above 80%. According to the standard parameters, the following kinetic patterns were defined: percentages of motile sperm and progressive motile sperm; fast, medium, and slow speed sperm; average path velocity (VAP), curvilinear velocity (VCL), straight-line velocity (VSL), the amplitude of lateral head displacement (ALH), beat/cross frequency (BCF), percentage of straightness (STR), of linearity (LIN), and WOB (mean value of the ratio between VAP and VCL). Twenty-five images/s were captured in 100× magnification; measurements were performed in a 24×24 mm cover slide with a 10 μL drop of each sample. A minimum of 500 spermatozoa per sample were evaluated in at least five different fields.

### Epifluorescence microscopy assessment

All analyses were performed with a protected filter for optimized reading at an epifluorescence microscope (Nikon Eclipse Ci- Nikon Corporation - Japan). The samples were distributed on a glass slide overlaid with a cover slip for each staining protocol and observed in 1000× magnification with oil immersion. At least 200 sperm cells were assessed and counted, except for the sperm-binding protocol. No background correction was applied due to cell counting of cells was performed immediately and in the same settings.

### Plasma membrane integrity

Membrane integrity was evaluated according to Alfradique et al. (2018), using two fluorescent probes that bind to nucleic acids: acridine orange (1:10,000) and propidium iodide (0.5 mg/mL). Acridine orange is permeable in viable cells and emits green fluorescence. Propidium iodide is permeable in nonviable cells and cells with compromised membranes and emits red fluorescence. The filter used was 515-555 nm emission.

### Sperm capacitation and acrosome reaction

A chlortetracycline (CTC) solution was prepared daily in a buffer containing 20 mM Tris, 130 mM NaCl, 0.75 mM CTC, and 5 mM cysteine, pH 7.8. For staining, 10 µL of sperm sample was mixed with 10 µL of CTC solution onto a glass slide and evaluated with an emission filter of 470 nm. The spermatozoa were classified into three patterns: F-pattern corresponds to non-capacitated and acrosome-intact spermatozoa (bright fluorescence over the whole head); B-pattern corresponds to capacitated and acrosome-intact spermatozoa (fluorescence-free band in the post-acrosomal region); and AR-pattern corresponds to acrosome-reacted spermatozoa (dull fluorescence over the whole head except for a thin, bright band of fluorescence along the equatorial region and in mid-piece (Olivares et al., 2017).

### Mitochondrial activity

MitoTracker Green was used for staining, with a final concentration of 20 µM. The evaluation was performed after incubation of semen (10 µL) and MitoTracker solution (10 µL) for 10 min at 37 °C, and an emission filter of 488 nm. The fluorochrome only labels the mitochondria with mitochondrial membrane potential (MMP) of live spermatozoa (Druart et al., 2009). Sperm were classified as high MMP (bright green fluorescence), low MMP (low green fluorescence), and inactive MMP (no green fluorescence observed).

### Sperm binding

The perivitelline membrane was obtained by separating the intact egg yolk from the albumen of non-fertile chicken eggs. The membrane was washed with PBS inside a petri dish covered with parafilm until all the yolk was removed. Then, the membrane was cut into squares of 0.5 cm^2^. The membrane was covered with 1 mL of FERT-TALP medium (0.33 g NaCl, 0.011 g KCl, 100 μL NaH_2_PO_4_, 93 μL Na lactate, 0.105 g NaHCO_3_, 100 μL Phenol Red, 0.0135 g caffeine, 0.0147 g CaCl_2_.2H_2_O, 50 μL MgCl_2_, 0.119 g HEPES) and 20 μL of sperm sample was added. The semen sample with the membrane was incubated for 1 h at 38.5 °C with 5% of CO_2_. After incubation, the membrane was washed with PBS to remove any unbound sperm, distributed on a glass slide, 1 μL of Hoechst 33342 (1 mg/mL) was added, and covered with a coverslip sealed with fingernail polish. Five fields were counted per sample, and the results of spermatozoa binding were expressed as mm^2^ of membrane (Barbato et al., 1998; Moraes et al., 2010).

### Hypo-osmotic assay and morphology

For the hypo-osmotic assay, aliquots of 30 μL of semen were placed in tubes with 1000 μL of Milli-Q water (0 mOsm) (Menezes et al., 2013). Then, these aliquots were incubated at 37 °C for 20 min, and 10 μL was distributed in a glass slide covered with a coverslip and evaluated in phase contrast microscopy at 1000x magnification with oil immersion (Ramu and Jeyendran, 2013). For morphology, a 30 μL sample of semen was added to 1000 μL of formol saline and stored at 4 °C until evaluation. Samples were distributed on a glass slide overlaid with a cover slip and sealed with fingernail polish. Abnormal sperm were classified as major and minor defects. At least 200 sperm cells were evaluated in both assays (CBRA, 2013).

### Lipoperoxidation

Aliquots of 500 μL of samples from each treatment and 1000 μL of 10% trichloroacetic acid solution (10% TCA) were centrifuged at 1800 *g* for 15 min at 15 °C for precipitation of proteins. Aliquots of 500 μL of the supernatant were placed in tubes along with 500 μL of 1% thiobarbituric acid, dissolved in 0.05 N sodium hydroxide, freshly prepared. The tubes were incubated in a water bath at 100 °C for 10 min and then cooled in ice to stop the reaction. Thiobarbituric Acid Reactive Species (TBARS) were quantified in a spectrophotometer, at a length of 532 nm. Each semen sample was evaluated in triplicates, and distilled water was used as a negative control. The results were compared with a standard MDA curve and expressed in ng/mL of TBARS per semen sample (Sarlós et al., 2002).

### Statistical analysis

Data analyses were performed in IBM SPSS version 25. Data were compared using a generalized linear mixed model (GLMM), including evaluation moment (before and after cryopreservation), AFP I, resveratrol, and their interactions as main effects in endpoints, with data of the different treatments nested to the pooled sample. In analyses carried out only after thawing, the GLMM included AFP I, resveratrol, and their interactions as main effects in endpoints. The Sidak test was used for post hoc comparisons. For all tests, differences were considered significant when *P* < 0.05, and as a high probability when 0.10 ≥ *P* > 0.05 (Ferreira and Patino, 2015), and data are presented as LSmeans (±SEM).

## Results

Immediately after dilution and before cryopreservation, no differences were observed in experimental treatments on sperm kinetics, plasma membrane integrity, hypo-osmotic test, and mitochondrial activity parameters immediately after dilution (Table 1). There was also no interaction between the moment of evaluation, AFP I, and resveratrol. However, thawing had significant effects on several variables evaluated (Table 1).

**Table 1:** Ram sperm endpoints after dilution (before freezing) and immediately after thawing in extenders containing (+) or not (-) antifreeze protein (AFP) type I (0.1 μg/mL), associated or not with different concentrations of resveratrol (0, 10, or 50 μM/mL) during cryopreservation (LSmeans ± SEM).

**Endpoints**	**AFP I** **(μg/mL)**	**Before cryopreservation**			**Frozen-Thawed**			***P-value***			
		**Resveratrol (μM/mL)**			**Resveratrol (μM/mL)**						
	**0.1**	**0**	**10**	**50**	**0**	**10**	**50**	**AFP I**	**Resveratrol**	**Evaluation moment**	**AFP×Resveratrol×Evaluation moment**
Total motility (%)	-	99.0 ± 4.0	99.4 ± 4.0	98.3 ± 4.0	25.3 ± 3.8	33.9 ± 3.8	24.3 ± 3.8	n.s.	n.s.	0.001	n.s.
	+	99.6 ± 4.0	99.1 ± 4.0	99.2 ± 4.0	28.7 ± 3.8	24.0 ± 3.8	23.9 ± 3.8				
VCL (µm/s)	-	74.3 ± 6.0	72.1 ± 6.0	66.9 ± 6.0	23.7 ± 6.0	24.1 ± 6.0	22.6 ± 6.0	n.s.	n.s.	0.001	n.s.
	+	74.2 ± 6.0	69.9 ± 6.0	69.4 ± 6.0	24.8 ± 6.0	25.1 ± 6.0	21.8 ± 6.4				
VSL (µm/s)	-	30.4 ± 2.5	27.1 ± 2.5	25.1 ± 2.5	15.5 ± 2.5	15.1 ± 2.5	13.2 ± 2.5	n.s.	n.s.	0.001	n.s.
	+	28.9 ± 2.5	28.3 ± 2.5	25.2 ± 2.5	15.3 ± 2.5	15.7 ± 2.5	13.9 ± 2.5				
VAP (µm/s)	-	46.2 ± 3.7	42.7 ± 3.7	39.7 ± 3.7	18.8 ± 3.7	18.6 ± 3.7	16.8 ± 3.7	n.s.	n.s.	0.001	n.s.
	+	45.7 ± 3.7	43.1 ± 3.7	40.7 ± 3.7	19.0 ± 3.7	19.5 ± 3.7	17.2 ± 3.7				
LIN (%)	-	37.8 ± 2.6	38.6 ± 2.4	36.3 ± 2.6	64.5 ± 2.4	62.1 ± 2.4	57.7 ± 2.4	n.s.	n.s.	0.001	n.s.
	+	38.7 ± 2.4	40.6 ± 2.4	37.1 ± 2.4	60.5 ± 2.4	62.3 ± 2.4	61.0 ± 2.4				
STR (%)	-	62.8 ± 1.9	64.1 ± 1.8	62.2 ± 1.9	81.6 ± 1.8	80.7 ± 1.8	77.8 ± 1.8	n.s.	n.s.	0.001	n.s.
	+	63.2 ± 1.8	65.4 ± 1.8	62.7 ± 1.8	79.5 ± 1.8	80.3 ± 1.8	80.5 ± 1.8				
WOB (%)	-	59.8 ± 1.8	59.7 ± 1.7	58.4 ± 1.8	78.9 ± 1.7	76.8 ± 1.7	73.8 ± 1.7	n.s.	n.s.	0.001	n.s.
	+	61.2 ± 1.7	61.5 ± 1.7	59.0 ± 1.7	75.8 ± 1.7	77.4 ± 1.7	75.7 ± 1.7				
ALH (µm)	-	3.3 ± 0.3	3.5 ± 0.3	3.4 ± 0.3	2.9 ± 0.3	3.4 ± 0.3	3.3 ± 0.3	n.s.	n.s.	0.001	n.s.
	+	4.0 ± 0.3	3.3 ± 0.3	3.8 ± 0.3	2.7 ± 0.3	2.7 ± 0.3	2.7 ± 0.3				
BCF (Hz)	-	7.7 ± 0.9	7.6 ± 0.9	7.5 ± 0.9	2.3 ± 1.0	1.9 ± 1.0	1.7 ± 1.0	n.s.	n.s.	0.001	n.s.
	+	7.2 ± 0.9	7.8 ± 0.9	7.8 ± 0.9	1.4 ± 1.0	1.2 ± 1.0	1.4 ± 1.0				
Plasma Membrane Integrity (%)	-	81.1 ± 2.3	81.1 ± 2.3	82.6 ± 2.3	24.1 ± 2.4	30.2 ± 2.3	28.7 ± 2.3	n.s.	n.s.	0.001	n.s.
	+	78.6 ± 2.7	81.3 ± 2.3	81.9 ± 2.3	27.5 ± 2.3	30.3 ± 2.3	27.9 ± 2.5				
Hypo-osmotic (%)	-	90.7 ± 2.1	92.9 ± 2.1	92.3 ± 2.1	19.7 ± 2.1	24.9 ± 2.1	22.4 ± 2.1	0.06	n.s.	0.001	n.s.
	+	91.1 ± 2.1	90.3 ± 2.1	91.3 ± 2.1	17.4 ± 2.1	18.9 ± 2.1	20.0 ± 2.1				
High MMP (%)	-	69.5 ± 3.5	69.5 ± 3.5	69.6 ± 4.2	23.6 ± 3.8	29.4 ± 3.5	23.3 ± 3.8	0.07	n.s.	0.001	n.s.
	+	72.5 ± 3.5	74.5 ± 3.5	71.8 ± 3.5	30.6 ± 3.5	25.3 ± 3.8	33.0 ± 3.5				
Low MMP (%)	-	19.5 ± 3.8	20.5 ± 3.5	20.0 ± 3.8	35.0 ± 3.5	35.9 ± 3.5	40.5 ± 3.8	n.s.	n.s.	0.001	n.s.
	+	22.0 ± 3.5	19.6 ± 3.5	17.8 ± 3.5	36.5 ± 3.5	37.0 ± 3.5	30.6 ± 3.8				
Inactive MMP (%)	-	6.9 ± 5.4	7.1 ± 5.4	9.0 ± 5.4	38.9 ± 5.0	34.7 ± 5.0	29.4 ± 5.4	n.s.	n.s.	0.001	n.s.
	+	6.4 ± 5.4	9.4 ± 5.0	10.3 ± 5.0	32.9 ± 5.0	34.6 ± 5.0	32.4 ± 5.0				

Abbreviations: VCL: curvilinear velocity; VSL: straight-line velocity; VAP: average path velocity; LIN: linearity; STR: straightness; WOB: wobble; ALH: amplitude of lateral head displacement; BCF: beat/cross-frequency; MMP: mitochondrial membrane potential; n.s.: non-significant.

In frozen-thawed semen, there were no significant effects on sperm kinetics, plasma membrane integrity, hypo-osmotic test, and mitochondrial activity parameters. However, the addition of AFP I shows a high probability of reducing the quantity of sperm with functional membrane percentage in the hypo-osmotic test (*P* = 0.06) and increasing the percentage of sperm with high mitochondrial activity (*P* = 0.07) (Table 1). On the other hand, the concentration of resveratrol did not affect any of these variables (Table 1).

**Figure 2:**
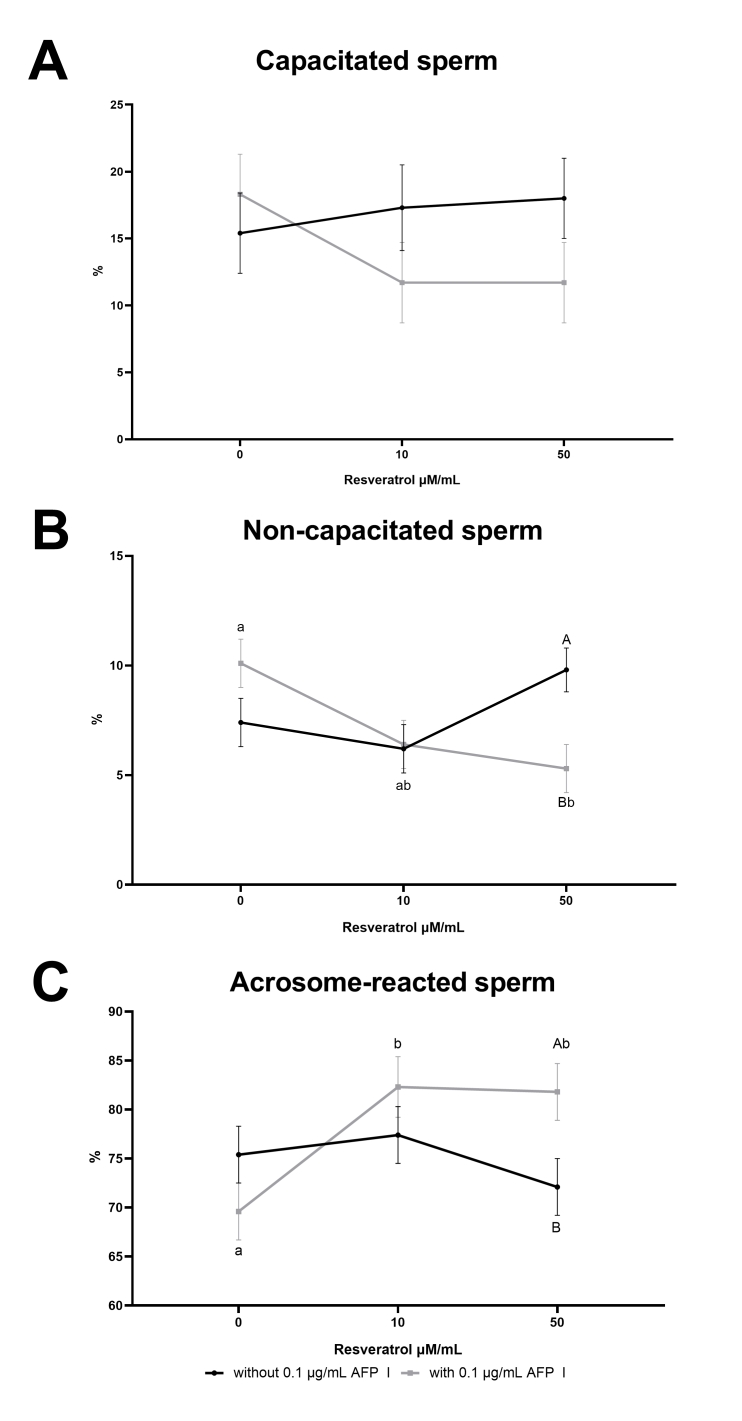
Interaction of (A) capacitated sperm, (B) non-capacitated sperm, and (C) acrosome-reacted sperm of frozen-thawed ram semen with or without the association of AFP I and different concentrations of resveratrol, immediately after thawing. Within a column or row, values with different superscripts differ significantly (*P* < 0.05). A,B differs between the absence or presence of AFP I (0.1 μg/mL). ^a,b^differs among resveratrol concentrations (0, 10, or 50 μM/mL).

There were significant interactions between AFP I and resveratrol in the percentage of non-capacitated sperm (*P* = 0.009) and sperm with acrosomal reaction (*P* = 0.034) (Figure 2B and 2C). This interaction reduced the percentage of non-capacitated sperm in the presence of AFP I and 50 μM/mL of resveratrol in the extender, an effect that was not observed in the absence of AFP I. The percentage of sperm with acrosomal reaction increased with the addition of AFP I and in the presence of 10 or 50 μM/mL of resveratrol, an effect that was not observed with AFP I without resveratrol. Moreover, this variable had a high probability of differences according to the resveratrol concentration factor (*P* = 0.057). There were no effects of the treatments or their interactions on the percentage of capacitated sperm.

**Figure 3:**
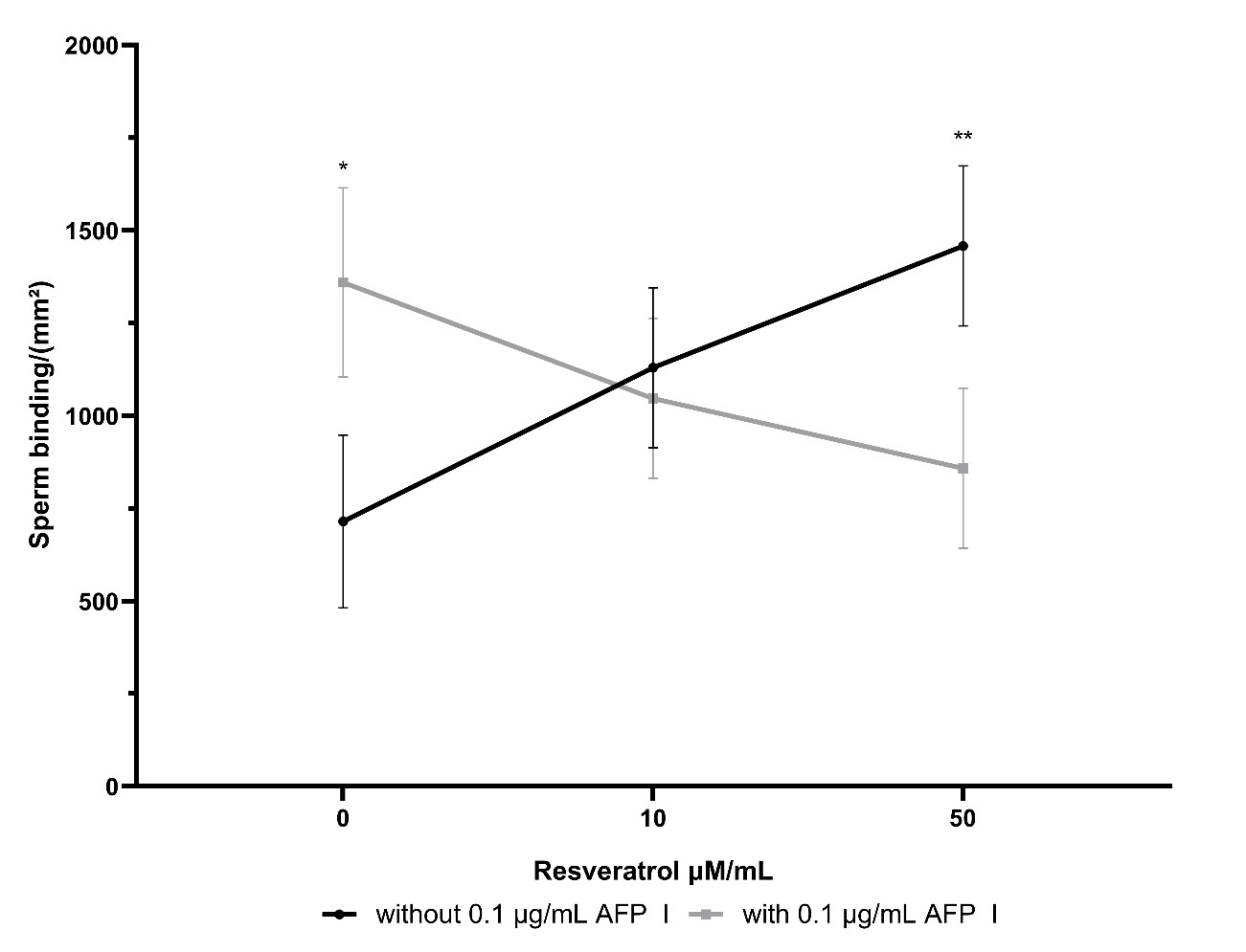
Interaction between the association of AFP I and resveratrol concentrations in semen extender on frozen-thawed ram sperm bound to egg perivitelline membrane test, immediately after thawing. *represents the high probability (*P* = 0.071) of sperm bound in a single addiction of 0.1 µg/mL of AFP I in interaction analysis; **represents the high probability (*P* = 0.058) of sperm bound in a single addiction of 50 µM/mL of resveratrol in the interaction analysis.

There was an interaction between AFP I and resveratrol in the percentage of sperm that bound to the egg perivitelline membrane (*P* = 0.04) (Figure 3). The addition of AFP I and resveratrol per se did not affect the percentage of spermatozoa bound to the perivitelline membrane. However, when added simultaneously, it led to a decrease in it. Nevertheless, no significant differences were observed if only either AFP I or resveratrol were added. Regarding peroxidation, there was no interaction (Table 2), although there was a high probability of increasing the concentration of TBARS by adding AFP I (*P*=0.085) (Table 2). Lastly, AFP I, resveratrol, or their interaction did not affect sperm morphology.

**Table 2:** Normal morphology and lipid peroxidation assessed by thiobarbituric acid reactive substances (TBARS) levels of cryopreserved ram sperm in extenders containing (+) or not (-) antifreeze protein type I (0.1 μg/mL), associated or not with different concentrations of resveratrol (0, 10 or 50 μM/mL) during cryopreservation (LSmeans ± SEM).

**Endpoints**	**AFP I (μg/mL)**	**Resveratrol (μM/mL)**			***P-value***		
	**0.1**	**0**	**10**	**50**	**AFP I**	**Resveratrol**	**AFP×Resveratrol**
Normal Morphology (%)	-	79.8 ± 2.1	77.8 ± 2.1	77.1 ± 2.3	n.s.	n.s.	n.s.
	+	81.7 ± 2.3	80.1 ± 2.6	79.5 ± 2.3			
TBARS (ng/mL)	-	477.2 ± 66.2	613.9 ± 60.5	534.7 ± 66.2	0.085	n.s.	n.s.
	+	568.2 ± 60.5	666.1 ± 60.5	664.6 ± 60.5			

Abbreviations: n.s.: non-significant.

## Discussion

In general, the results of this study showed that the association of AFP I with resveratrol for cryopreservation of ram semen did not generate positive results, and there were even inferior results when the concentration of resveratrol increased. Conversely, the addition of 50 μM/mL of resveratrol or 0.1 μg/mL of AFP I, without associating with each other, led to a higher sperm binding, suggesting that both can improve the fertilizing capacity. Therefore, adding both molecules was disadvantageous for sperm cryopreservation.

The addition of AFP I had no positive effects on fresh semen. However, it should be expected that the main effects of adding AFP I would be in thawed sperm, as it avoids the formation of ice crystals. In this sense, the lack of positive results differs from a previous study performed by the same group (Correia et al., 2021a), where the addition of AFP I increased the percentage of sperm with intact membranes, improved the LIN and STR rate in the kinetic parameters, and increased the percentage of normal sperm. Moreover, in the present study, the addition of AFP I induced an increase in mitochondrial activity, and plasma membrane functionality decreased, after cryopreservation. The semen collection was the main methodological difference between both studies as Correia et al. (2021a) used electroejaculation, while in the present study, an artificial vagina was used. Of note, the same extender composition, as well as freezing and thawing protocols, were applied in both studies. According to Ledesma et al. (2015), the ram sperm collected by electroejaculation is more resistant to cryopreservation than that collected by an artificial vagina. This may be explained by the greater amount of seminal plasma proteins in the semen collected by electroejaculation, which could increase sperm cryoresistance (Marco-Jiménez et al., 2005; Ledesma et al., 2015). Moreover, the addition of seminal plasma proteins associated with membrane protectors, such as vitamin E, increases sperm viability after heat shock (Pérez-Pé et al., 2001). In *Macaca fascicularis*, the addition of 0.1 µg/mL of AFP III in semen extender significantly reduced the number of differential proteins in cryopreserved compared to fresh spermatozoa (Chen et al., 2021). Thus, the greater amount of seminal plasma might have potentiated the cryoprotective effect of AFP I. In the study conducted by Correia et al. (2021a), the addition of 0.1 μg/mL of AFP I increased the percentage of normal sperm after cryopreservation, while in the present study, there was no statistical difference between the AFP I group and the control, and this difference that can be explained by the facts mentioned above. However, to confirm this possible action, it would be required to compare the effects of AFP I in sperm collected with both techniques in a single study.

Resveratrol is known to prevent premature acrosomal reaction (Silva et al., 2012), which is consistent with the greater percentage of non-capacitated sperm observed in this study when only 50 µM/mL of resveratrol was added to the semen extender. Similarly, this concentration reduced the cryo-induced capacitation in buffalo sperm (Longobardi et al., 2017). On the other hand, AFP I alone did not produce this effect on capacitation status and acrosomal reaction. In the previous report conducted by Correia et al. (2021a), no differences were observed in the acrosome integrity pattern, suggesting that the addition of AFP does not affect capacitation during cryopreservation. When evaluating the addition of both AFP I and resveratrol, an increase in the percentage of acrosomal reaction and a reduced percentage of non-capacitated sperm was observed. This phenomenon could be related to calcium influx leading to cellular alterations during cryopreservation (Watson, 2000) or by molecular destabilization in sperm; however, the cryocapacitation mechanisms are not fully elucidated (Benko et al., 2022).

The egg perivitelline membrane displays homology to mammalian ZP3 and, thus, the binding test is closely related to the sperm fertilizing capacity (Losano et al., 2015). This technique has already been demonstrated to present a linear relationship with *in vivo* fertility in roosters (Barbato et al., 1998), high sensibility for *in vitro* penetration assay in Vesper Mice (Corcini et al., 2012), and positive correlations for acrosomal and membrane integrity, mitochondrial activity, and motility in bulls (Losano et al., 2015) and dogs (Brito et al., 2017). The use of resveratrol at 50 µM/mL concentration associated with AFP I decreased the binding rate to the egg perivitelline membrane. Compared to the control group, the addition of 50 µM/mL resveratrol alone did not have any deleterious effect and led to an increase in sperm binding. In Buffalo, the supplementation of 50 µM/mL of resveratrol was able to increase the *in vitro* fertilization ability but did not affect the *in vivo* outcomes (Longobardi et al., 2017), while 100 µM/mL of resveratrol was able to increase *in vivo* pregnancy (Ahmed et al., 2020). Moreover, the use of AFP I alone promoted a similar higher amount of sperm bounded. However, it is still necessary to test these treatments on *in vivo* conditions to determine the potential of these molecules in artificial insemination. There was also a high probability of TBARS level to increase after AFP I addition in the semen extender. This deleterious response could be related to a decrease in plasma membrane functionality. These results indicate that 50 µM/mL of resveratrol without association with AFP could be able to increase the fertilizing capacity of ram-frozen sperm and maintain both TBARS levels and plasma membrane functionality. In goat semen, resveratrol supplementation did not affect the TBARS concentration, similar to that observed in the current study (Falchi et al., 2020). In the sheep study by Al-Mutary et al. (2020), the addition of resveratrol to cooled/chilled semen increased sperm motility and increased the blastocyst rate. In human, the addition of resveratrol also did not affect any analyzed sperm parameters but led to a significant decrease in the DNA fragmentation rate (Nashtaei et al., 2017). Resveratrol can activate the 5' adenosine monophosphate-activated protein kinase protein (AMPK) (Pasquariello et al., 2020) by resveratrol-induced ROS reduction in cryopreserved human semen samples (Branco et al., 2010; Nashtaei et al., 2017). In boar, an improvement in the quality of cryopreserved semen was identified by the resveratrol-activated AMPK pathway (Zhu et al., 2019), reducing DNA damage, increasing mitochondrial activity, and reducing ROS levels, among other factors evaluated. Thus, although the AMPK pathway was not assessed in the present study, the increase in binding to the perivitelline membrane may have occurred due to acrosome maintenance and to the lower genetic material damage induced by ROS, as in other studies reported above.

The effect of antioxidant addition in semen extender differs according to the species, medium, and antioxidant type and concentration (Silva et al., 2012), and consistent advantages are still unclear and require elucidation. It is known that resveratrol has multiple interesting properties for distinct therapeutic activities, such as antioxidant, anti-inflammatory, neuroprotective, and anticarcinogenic effects, but it is a molecule with very low oral bioavailability due to low water solubility and stability (Atanacković et al., 2012), despite its high permeability (Walle, 2011). Of note, strategies to overcome the limitations of resveratrol solubility are still being investigated (Ghazwani et al., 2021). Regarding the negative effect of resveratrol and AFP association, a possible explanation for this unexpected result may be related to their biochemical and physicochemical molecules. While resveratrol can bind to proteins and change their functions mainly at hydrophobic sites in the tertiary structure (Gorji et al., 2015; Wu et al., 2020), AFPs bind to ice crystals due to their hydrophobic sites (Gharib et al., 2022), therefore competing at the site of action and inhibiting the internalization of resveratrol into the cell, resulting in a deleterious effect when associated. The interaction of resveratrol with the alpha-helix of AFP I could induce the inactivation of protein motifs and the function of AFP, influencing the antioxidant activity of resveratrol (Sun et al., 2022), leading to the formation of insoluble complexes (Bandyopadhyay et al., 2012).

**Figure 4:**
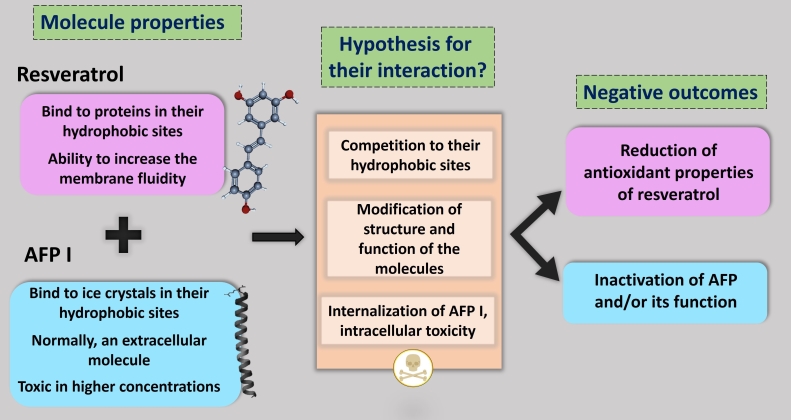
A diagrammatic figure summarizing the main hypothesis explaining why the association between antifreeze protein I (AFP I) and resveratrol did not promote benefits for ram sperm cryopreservation.

As already stated, AFP I can protect the membrane cells as an extracellular cryoprotectant when the correct concentrations are applied but induce deleterious effects when used in higher concentrations. Reducing the freezing point below the melting point and changing the crystal shapes efficiently inhibit ice recrystallization when AFP is used (see review Correia et al., 2021b). Meanwhile, resveratrol can increase the membrane fluidity caused by disturbances in the activity of transmembrane proteins (Płachta et al., 2024). However, when both molecules were associated, adverse effects were detected. Our main hypothesis to explain this harmful interaction is based on the fact that both resveratrol and AFP I have hydrophobic sites; thus, a possible competition between them could inhibit the internalization of the former into the cell. Considering the resveratrol's ability to modify membrane permeability, it cannot be ruled out that AFP I – typically an extracellular cryoprotectant used in low concentrations to avoid cytotoxicity – could have penetrated the sperm, leading to intracellular toxicity (Figure 4). Although those explanations appear consistent, they should be confirmed with future studies designed specifically to test them. It is reasonable to indicate, however, that the use of both molecules simultaneously during cryopreservation appears to be a non-recommended option. Even though the outcomes of this association were unexpected and, why not disappointing somehow, undesirable results are extremely useful in providing valuable insights to guide future research, thereby contributing substantially to the field.

## Conclusion

The association of resveratrol and AFP I did not improve the quality of frozen-thawed ram semen and induced some deleterious effects compared to the single addition of each one in the semen extender. The supplementation of 50 µM/mL of resveratrol alone improved the non-capacitated sperm and fertilizing capacity with no adverse effect on lipoperoxidation or sperm viability, being a potential cryoprotectant for ram sperm.

## References

[B001] Ahmed H, Jahan S, Ullah H, Ullah F, Salman MM (2020). The addition of resveratrol in tris citric acid extender ameliorates post-thaw quality parameters, antioxidant enzymes levels, and fertilizing capability of buffalo (*Bubalus bubalis*) bull spermatozoa. Theriogenology.

[B002] Alfradique VAP, Batista RITP, Souza-Fabjan JMG, Côrtes LR, Bragança GM, Souza CV, Costa LC, Brandão FZ (2018). Supplementation of 17β-estradiol and progesterone in the co-culture medium of bovine oviductal epithelial cells and ovine spermatozoa reduces the sperm kinematics and capacitation. Reprod Biol.

[B003] Al-Mutary MG, Al-Ghadi MQ, Ammari AA, Al-Himadi AR, Al-Jolimeed AH, Arafah MW, Amran RA, Aleissa MS, Swelum AA (2020). Effect of different concentrations of resveratrol on the quality and *in vitro* fertilizing ability of ram semen stored at 5 °C for up to 168 h. Theriogenology.

[B004] Arav A, Saragusty J, Niemann H, Wrenzycki C (2018). Animal biotechnology..

[B005] Atanacković MT, Gojković-Bukarica LC, Cvejić JM (2012). Improving the low solubility of resveratrol. BMC Pharmacol Toxicol.

[B006] Bandyopadhyay P, Ghosh AK, Ghosh C (2012). Recent developments on polyphenol-protein interactions: effects on tea and coffee taste, antioxidant properties and the digestive system. Food Funct.

[B007] Barbato GF, Cramer PG, Hammerstedt RH (1998). A practical *in vitro* sperm-egg binding assay that detects subfertile males. Biol Reprod.

[B008] Benko F, Mohammadi-Sangcheshmeh A, Ďuračka M, Lukáč N, Tvrdá E (2022). *In vitro* versus cryo-induced capacitation of bovine spermatozoa, part 1: Structural, functional, and oxidative similarities and differences. PLoS One.

[B009] Branco CS, Garcez ME, Pasqualotto FF, Erdtman B, Salvador M (2010). Resveratrol and ascorbic acid prevent DNA damage induced by cryopreservation in human semen. Cryobiology.

[B010] Brito MM, Lúcio CF, Angrimani DSR, Losano JDA, Dalmazzo A, Nichi M, Vannucchi CI (2017). Comparison of cryopreservation protocols (single and two-steps) and thawing (fast and slow) for canine sperm. Anim Biotechnol.

[B011] Chen B, Wang S, Inglis BM, Ding H, Suo A, Qiu S, Duan Y, Li X, Li S, Sun WQ, Si W (2021). Improving sperm cryopreservation with type III antifreeze protein: proteomic profiling of Cynomolgus Macaque (*Macaca fascicularis*) sperm. Front Physiol.

[B012] CBRA (2013). Manual para exame andrológico e avaliação de sêmen animal..

[B013] Corcini CD, Stephan MH, Colares EP, Santos EC, Varela AS, Bongalhardo DC, Lucia T (2012). In vitro assays for vesper mice (*Calomys laucha*) sperm using heterologous substrates from nonrodent species. J Exp Zool Part A Ecol Genet Physiol.

[B014] Correia LFL, Alves BRC, Batista RITP, Mermillod P, Souza-Fabjan JMG (2021). Antifreeze proteins for low-temperature preservation in reproductive medicine: a systematic review over the last three decades. Theriogenology.

[B015] Correia LFL, Espírito-Santo CG, Braga RF, Carvalho-de-Paula CJC, da Silva AA, Brandão FZ, Freitas VJF, Ungerfeld R, Souza-Fabjan JMG (2021). Addition of antifreeze protein type I or III to extenders for ram sperm cryopreservation. Cryobiology.

[B016] Druart X, Cognié J, Baril G, Clément F, Dacheux JL, Gatti JL (2009). *In vivo* imaging of *in situ* motility of fresh and liquid stored ram spermatozoa in the ewe genital tract. Reproduction.

[B017] Falchi L, Pau S, Pivato I, Bogliolo L, Zedda MT (2020). Resveratrol supplementation and cryopreservation of buck semen. Cryobiology.

[B018] Ferreira JC, Patino CM (2015). What does the p value really mean?. J Bras Pneumol.

[B019] Gharib G, Saeidiharzand S, Sadaghiani AK, Koşar A (2022). Antifreeze proteins: a tale of evolution from origin to energy applications. Front Bioeng Biotechnol.

[B020] Ghazwani M, Alam P, Alqarni MH, Yusufoglu HS, Shakeel F (2021). Solubilization of *trans*-resveratrol in some mono-solvents and various propylene glycol + water mixtures. Molecules.

[B021] Gorji EG, Rocchi E, Schleining G, Bender-Bojalil D, Furtmüller P, Piazza L, Iturri JJ, Toca-Herrera JL (2015). Characterization of resveratrol-milk protein interaction. J Food Eng.

[B022] Gualtieri R, Kalthur G, Barbato V, Longobardi S, Di Rella F, Adiga SK, Talevi R (2021). Sperm oxidative stress during *in vitro* manipulation and its effects on sperm function and embryo development. Antioxidants.

[B023] Harikumar KB, Aggarwal BB (2008). Resveratrol: A multitargeted agent for age-associated chronic diseases. Cell Cycle.

[B024] Jha PK, Shahi Alam MG, Mansur AAL, Naher N, Islam T, Bhuiyan MU, Bari FY (2019). Cryopreservation of Bangladeshi ram semen using different diluents and manual freezing techniques. Cryobiology.

[B025] Kim HJ, Lee JH, Hur YB, Lee CW, Park SH, Koo BW (2017). Marine antifreeze proteins: structure, function, and application to cryopreservation as a potential cryoprotectant. Mar Drugs.

[B026] Ledesma A, Manes J, Ríos G, Aller J, Cesari A, Alberio R, Hozbor F (2015). Effect of seminal plasma on post-thaw quality and functionality of *Corriedale* ram sperm obtained by electroejaculation and artificial vagina. Reprod Domest Anim.

[B027] Longobardi V, Zullo G, Salzano A, De Canditiis C, Cammarano A, De Luise L, Puzio MV, Neglia G, Gasparrini B (2017). Resveratrol prevents capacitation-like changes and improves in vitro fertilizing capability of buffalo frozen-thawed sperm. Theriogenology.

[B028] Losano JD, Angrimani DS, Pereira RJ, Rocha AM, Criscuolo TS, Barnabe VH, Barnabe RC, Mendes CM, Assumpção ME, Nichi M (2015). Utilisation of sperm-binding assay combined with computer-assisted sperm analysis to evaluate frozen-thawed bull semen. Andrologia.

[B029] Lv C, Wu G, Hong Q, Quan G (2019). Spermatozoa cryopreservation: state of art and future in small ruminants. Biopreserv Biobank.

[B030] Lv C, Larbi A, Wu G, Hong Q, Quan G (2019). Improving the quality of cryopreserved goat semen with a commercial bull extender supplemented with resveratrol. Anim Reprod Sci.

[B031] Marco-Jiménez F, Puchades S, Gadea J, Vicente JS, Viudes-de-Castro MP (2005). Effect of semen collection method on pre- and post-thaw Guirra ram spermatozoa. Theriogenology.

[B032] Menezes GFO, Bittencourt RF, Ribeiro AL, Chalhoub M, Bittencourt MF, Oba E, Bicudo SD (2013). Utilization of the osmotic shock to assess the frozen ram semen viability. Braz J Vet Res Anim Sci.

[B033] Moraes EA, Graham JK, Torres CAA, Meyers M, Spizziri B (2010). Delivering cholesterol or cholestanol to bull sperm membranes improves cryosurvival. Anim Reprod Sci.

[B034] Nashtaei MS, Amidi F, Gilani MAS, Aleyasin A, Bakhshalizadeh S, Naji M, Nekoonam S (2017). Protective features of resveratrol on human spermatozoa cryopreservation may be mediated through 5'AMP-activated protein kinase activation. Andrology.

[B035] Nishijima K, Tanaka M, Sakai Y, Koshimoto C, Morimoto M, Watanabe T, Fan J, Kitajima S (2014). Effects of type III antifreeze protein on sperm and embryo cryopreservation in rabbit. Cryobiology.

[B036] Olivares CCS, Souza-Fabjan JMG, Fonseca JF, Balaro MFA, Freitas VJF, Oliveira RV, Brandão FZ (2017). Comparison of different sperm selection techniques in ram frozen-thawed sperm. Acta Sci Vet.

[B037] Pasquariello R, Verdile N, Brevini TAL, Gandolfi F, Boiti C, Zerani M, Maranesi M (2020). The role of resveratrol in mammalian reproduction. Molecules.

[B038] Payne SR, Oliver JE, Upreti GC (1994). Effect of antifreeze proteins on the motility of ram spermatozoa. Cryobiology.

[B039] Pérez-Pé R, Cebrián-Pérez JA, Muiño-Blanco T (2001). Semen plasma proteins prevent cold-shock membrane damage to ram spermatozoa. Theriogenology.

[B040] Peris SI, Bilodeau JF, Dufour M, Bailey JL (2007). Impact of cryopreservation and reactive oxygen species on dna integrity, lipid peroxidation, and functional parameters in ram sperm. Mol Reprod Dev.

[B041] Płachta L, Mach M, Kowalska M, Wydro P (2024). The effect of trans-resveratrol on the physicochemical properties of lipid membranes with different cholesterol content. Biochim Biophys Acta Biomembr.

[B042] Qadeer S, Khan MA, Shahzad Q, Azam A, Ansari MS, Rakha BA, Ejaz R, Husna AU, Duman JG, Akhter S (2016). Efficiency of beetle (*Dendroides canadensis*) recombinant antifreeze protein for buffalo semen freezability and fertility. Theriogenology.

[B043] Ramu S, Jeyendran RS (2013). The hypo-osmotic swelling test for evaluation of sperm membrane integrity. Methods Mol Biol.

[B044] Sarlós P, Molnar A, Kokai M, Gábor G, Rátky J (2002). Comparative evaluation of the effect of antioxidants in the conservation of ram semen. Acta Vet Hung.

[B045] Silva ECB, Cajueiro JFP, Silva SV, Soares PC, Guerra MMP (2012). Effect of antioxidants resveratrol and quercetion on *in vitro* evaluation of frozen ram semen. Theriogenology.

[B046] Sun X, Sarteshnizi RA, Udenigwe CC (2022). Recent advances in protein-polyphenol interactions focusing on structural properties related to antioxidant activities. Curr Opin Food Sci.

[B047] Upadhyay VR, Ramesh V, Dewry RK, Kumar G, Raval K, Patoliya P (2021). Implications of cryopreservation on structural and functional attributes of bovine spermatozoa: an overview. Andrologia.

[B048] Walle T (2011). Bioavailability of resveratrol. Ann N Y Acad Sci.

[B049] Watson PF (2000). The causes of reduced fertility with cryopreserved semen. Anim Reprod Sci.

[B050] Wu Y, Hsieh TC, Wu JM, Wang X, Christopher JS, Pham AH, Swaby JD, Lou L, Xie ZR (2020). Elucidating the inhibitory effect of resveratrol and its structural analogs on selected nucleotide-related enzymes. Biomolecules.

[B051] Zhu Z, Li R, Fan X, Lv Y, Zheng Y, Hoque SAM, Wu D, Zeng W (2019). Resveratrol improves boar sperm quality via 5'AMP-activated protein kinase activation during cryopreservation. Oxid Med Cell Longev.

